# Development and Validation of a Pediatric Hospital-Acquired Malnutrition (PHaM) Risk Score to Predict Nutritional Deterioration in Hospitalized Pediatric Patients: A Secondary Analysis Based on a Multicenter Prospective Cohort Study

**DOI:** 10.3390/nu16172898

**Published:** 2024-08-29

**Authors:** Suchaorn Saengnipanthkul, Prapassara Sirikarn, Nalinee Chongviriyaphan, Narumon Densupsoontorn, Jeeraparn Phosuwattanakul, Amnuayporn Apiraksakorn, Phanthila Sitthikarnkha, Leelawadee Techasatian, Rattapon Uppala, Pagakrong Lumbiganon

**Affiliations:** 1 Department of Pediatrics, Faculty of Medicine, Srinagarind Hospital, Khon Kaen University, Khon Kaen 40002, Thailand; suchsa@kku.ac.th (S.S.); puntsi@kku.ac.th (P.S.); leelawadee@kku.ac.th (L.T.); rattapon@kku.ac.th (R.U.); paglum@kku.ac.th (P.L.); 2 Department of Epidemiology and Biostatistics, Faculty of Public Health, Khon Kaen University, Khon Kaen 40002, Thailand; 3 Division of Nutrition, Department of Pediatrics, Faculty of Medicine Ramathibodi Hospital, Mahidol University, Bangkok 10400, Thailand; nalinee.cho@mahidol.ac.th (N.C.); jeeraparn.pho@mahidol.ac.th (J.P.); 4 Division of Nutrition, Department of Pediatrics, Faculty of Medicine Siriraj Hospital, Mahidol University, Bangkok 10700, Thailand; narumon.den@mahidol.ac.th; 5 Department of Pediatrics, Khon Kaen Hospital, Khon Kaen 40000, Thailand; a.apiraksakorn@cpird.in.th

**Keywords:** predictive score, hospital-acquired malnutrition, nutritional deterioration, children, validation

## Abstract

(1) Background: Hospital-acquired malnutrition in pediatric patients leads to adverse outcomes. This study aimed to develop and validate a pediatric hospital-acquired malnutrition (PHaM) risk score to predict nutritional deterioration. (2) Methods: This was a derivative retrospective cohort study for developing a PHaM risk score. The study included data from children aged 1 month–18 years admitted to pediatric wards in four tertiary care hospitals for at least 72 h between December 2018 and May 2019. Data on pediatric patients’ characteristics, medical history, nutritional status, gastrointestinal symptoms, and outcomes were used for tool development. Logistic regression identified risk factors for nutritional deterioration, defined as a decline in BMI z-score ≥ 0.25 SD and/or ≥2% weight loss. A PHaM risk score was developed based on these factors and validated with an independent prospective cohort from July 2020 to March 2021. (3) Results: The study used a derivative cohort of 444 patients and a validation cohort of 373 patients. Logistic regression identified gastrointestinal symptoms, disease severity, fever, lower respiratory tract infection, and reduced food intake as predictors. The PHaM risk score (maximum 9 points) showed good discrimination and calibration (AUC 0.852, 95% CI: 0.814–0.891). Using a cut-off at 2.5 points, the scale had 63.0% sensitivity, 88.6% specificity, 76.1% positive predictive value, and 80.6% negative predictive value (NPV) when applied to the derivative cohort. The accuracy improved on the validation cohort, with 91.9% sensitivity and 93.0% NPV. (4) Conclusions: This PHaM risk score is a novel and probably effective tool for predicting nutritional deterioration in hospitalized pediatric patients, and its implementation in clinical practice could enhance nutritional care and optimize outcomes.

## 1. Introduction

Hospital-acquired malnutrition (HaM) or nutritional deterioration in children refers to a decline in nutritional status during hospitalization, regardless of their initial nutritional state. The prevalence of HaM in children varies across different healthcare settings and populations. Research indicates that HaM affects pediatric patients globally, with prevalence rates ranging from 19–60% worldwide [1,2,3,4,5,6]. Approximately 24% of hospitalized Thai children developed HaM, and it was associated with longer hospital stays and an increased risk of developing hospital-acquired infection [6]. Factors associated with HaM include young age (<24 months or <5 years), fever, gastroenteritis, nighttime abdominal pain, seizures, pneumonia, a moderate to severe grade of illness, a history of weight loss or poor weight gain, reduced food intake, and duration of hospital [2,4,5,6,7,8,9,10]. A decline in nutritional status can also result from nutritional deterioration during hospitalization. Hospital-acquired malnutrition is a condition that is underdiagnosed and undertreated, but it is made worse by factors such as mealtime disruptions, poor appetite, underlying medical conditions, hospital environments, treatment interventions, and nutritional support practices. These findings highlight the importance of early identification through the development of screening tools that are sensitive and specific to the pediatric population’s needs. Consequently, routine nutritional screening, assessment, and early intervention are crucial to prevent and to manage HaM effectively. Accordingly, the risks of complication in the patients are reduced and their health outcomes are improved.

The existing nutrition screening tools mainly focus on identifying malnutrition upon admission and require a health professional with anthropometric skills, which can be time-consuming. The complexity of these tools could be a barrier to their routine use. An ideal nutrition screening tool should have high sensitivity, specificity, validity, and reliability. It should be simple and easy to implement without extensive training or a specialist. In addition, it should offer quick, inexpensive, noninvasive clinical utility and be specifically catered to a pediatric inpatient population, including infants and children with certain clinical diagnoses [11]. This study aimed to develop and validate a quick and simple screening tool to identify HaM in hospitalized pediatric patients in tertiary care hospitals.

## 2. Materials and Methods

The study was conducted in three steps as follows: step 1, the derivative cohort, involved categorizing the study participants based on the occurrence of HaM; step 2 focused on developing and evaluating the model; and step 3 consisted of validating the model using a separate cohort.

### 2.1. Step 1: Derivative Cohort

The derivative group was conducted as a secondary analysis of data from a previous prospective, multicenter cohort study investigating the prevalence and risk factors for pediatric hospital-acquired malnutrition. The cohort study involved children aged 1 month to 18 years admitted to pediatric wards between December 2018 and May 2019. Nutritional status and clinical course were assessed at admission and weekly during hospitalization until discharge, using standard anthropometric measurements and laboratory tests to monitor patients’ nutritional status [6].

The derivative secondary analysis included pediatric patients hospitalized for over 72 h. Exclusions encompassed those lacking admission weight or height records; those with unstable conditions, infants under one month of corrected age; obese patients admitted solely for weight loss; and those presenting severe dehydration, organomegaly, significant masses or edema. Additionally, patients rehospitalized within one month were excluded. Patient characteristics were gathered from a cohort study, including age, sex, birth date, admission date, pre-existing conditions, current admission issues, weight, height, in-hospital comorbidities, and complications. Based on theoretical frameworks and prior studies, key variables potentially associated with PHaM risk were identified, including demographic, clinical, and nutritional factors.

We categorized diseases by severity (mild, moderate, or severe) as detailed in previous study’s criteria [8]. For example, acute gastroenteritis is categorized as mild, while acute fever in a patient with SLE is categorized as moderate. This approach helped to address the complexity of different disease etiologies within the practical constraints of a screening tool. Nutritional status was evaluated using anthropometric measurements, which were then converted to weight-for-age z-score (WAZ), length/height-for-age z-score (LAZ/HAZ), and body mass index z-score (BMIZ) for age and sex based on the World Health Organization (WHO) reference using the WHO Anthro software version 3.2.2 (children under 5 years old) or the WHO AnthroPlus software version 1.0.4 (children 5–18 years) [12].

Furthermore, in-hospital comorbidities and complications were documented along with specific details regarding nutritional interventions. Hospital-acquired infection pertains to any systemic or localized infection occurring 48 h after admission resulting from a response by an infectious agent or toxin. Types of nosocomial infection include bloodstream infection (septicemia), central line-associated bloodstream infections (CLABSIs), respiratory infection, surgical wound infection, diarrhea, antibiotic-associated colitis (AAC), clinical sepsis or fever with negative blood culture, and urinary infection [13]. Nutritional intervention is described as a provision of oral nutritional supplements with a medical formula or specialized formulation, enteral nutrition (nutrient delivery through feeding tubes to the gastrointestinal tract), or parenteral nutrition (an intravenous administration of nutrients) (Supplementary Materials).

The primary outcome, as HaM, was a reduction in body mass index Z-score (BMIZ) ≥ 0.25 SD and/or weight loss ≥ 2% of reference weight during hospitalization or upon discharge. In the derivative cohort, an initial analysis was conducted using both univariable and multivariable logistic regression models to investigate a potential relationship between demographic and medical condition variables and the occurrence of HaM (defined here as *p* < 0.25). Descriptive statistics on the patients’ characteristics and medical conditions were presented as the frequency and percentage for categorical data. Continuous data underwent assessment for normality using the Shapiro–Wilk test, with results presented as the median and interquartile range if the assumption of normality was not met.

### 2.2. Step 2: Model Development

In the model development phase, we conducted an initial evaluation of predictors and pediatric hospital-acquired malnutrition (PHaM) using simple logistic regression. The predictors with *p*-values less than 0.25 in the crude analysis were included in the initial model. The backward stepwise method was employed to select predictors for the final model. The PHaM risk score was created using a scoring system based on regression coefficients derived from the final model. The risk score was computed by dividing the coefficients by the smallest coefficient in the model and then rounding to the nearest integer (Beta/Sullivan) [14,15]. The optimal cut-off point was identified using the Youden index (Youden index = sensitivity + specificity − 1) [16]. The performance of the developed risk score model was assessed from two perspectives. Firstly, the model’s ability to distinguish between patients who did or did not develop HaM (model discrimination) was evaluated using the area under the receiver operating characteristic curve (AUC), along with sensitivity, specificity, positive predictive value, negative predictive value, likelihood ratio positive (LR+), and likelihood ratio negative (LR−). Secondly, the agreement between the PHaM prediction risk score and the observed value (calibration) was demonstrated by the ratio of expected to observed outcomes (E:O ratio) and Hosmer–Lemeshow goodness of fit. Internal validation was performed using the bootstrapping technique with 1000 replications to assess its stability and performance within the study cohort.

### 2.3. Step 3: Validation Cohort

For external validation, an independent prospective cohort of pediatric patients aged 1 month to 18 years, who were admitted to Srinagarind Hospital at Khon Kaen University from July 2020 to March 2021 with inclusion and exclusion criteria similar to those in the derivative cohort, was used for validating the risk score. Evaluations such as AUC, sensitivity, specificity, positive predictive value, and negative predictive value were conducted on this validation cohort to assess the performance of the risk score. To improve understanding of the PHaM risk score’s ability to predict HaM, a second sensitivity analysis was conducted among patients who did not receive nutritional intervention from either a pediatric nutritional specialist or a primary physician upon admission or within 72 h under specific circumstances. These circumstances included patients scheduled for pre-operative nutritional intervention, those with severe acute malnutrition or intestinal malabsorption, and cases where there was physician concern about malnutrition.

### 2.4. Ethical Considerations

This study was conducted following the ethical principles outlined in the Declaration of Helsinki. Ethical approval was obtained from the Institutional Review Board of Khon Kaen University Ethics Committee for Human Research (No.: HE641639) and was approved by the institutional review boards of all involved institutes. This secondary analysis utilized existing research data (No: HE611373), which were de-identified before being provided to the statistician, ensuring that participant identities could not be revealed through the outcomes of the analysis. Written informed consent for the validation cohort (No: HE631238) was obtained from parents or legal guardians of participating children.

### 2.5. Statistical Analysis

Data analysis was performed using STATA 15.0 (College Station, TX, USA). Descriptive statistics was used to summarize baseline characteristics as the number and frequency for categorical data and the mean and standard deviation (SD) or median and interquartile range for continuous data. A logistic regression model was employed in identifying risk factors and developing the mentioned risk score. The statistical performance of the PHaM risk prediction model was evaluated using the AUC for discrimination, as well as the E:O ratio and Hosmer–Lemeshow goodness of fit for calibration assessment.

Sample size estimation was calculated using R program 4.2.2 package “pmsamsize” and based on the prevalence from a previous study, 5 variables with 7 candidate predictive parameters, and expected model performance to show the optimism-adjusted C-statistic of 0.8. The minimum sample size required for new model development based on calculation was 281 with 68 events of HaM and an event per predictor parameter (EPP) of 9.63.

## 3. Results

### 3.1. Participant Characteristics

A total of 817 patients were included in the final analysis, with 444 in the derivative cohort and 373 in the validation cohort. The median age was consistent across both cohorts. However, the validation group had significantly higher percentages of female participants, patients admitted with more severe medical conditions, and participants with decreased food intake as well as more underweight and stunted participants than the derivative cohort, as shown in Table 1. The underlying conditions of the patients in our study are described in the Supplementary Materials.

The prevalence of hospital-acquired malnutrition (HaM) was found to be 36.5% in the derivative cohort and 39.7% in the validation cohort. Patients with HaM were more likely than those without HaM to have moderate to severe disease upon admission, experience gastrointestinal symptoms and fever, and have a history of weight loss. Furthermore, patients diagnosed with HaM faced a significantly higher risk of nosocomial infection and prolonged hospital stays than their counterparts without this condition. Nutritional intervention was prescribed for 47 (10.6%) individuals in the derivative cohort and 104 (27.9%) individuals in the validation cohort, reflecting a significantly higher rate in the validation group.

### 3.2. Model Development

#### The Pediatric Hospital-Acquired Malnutrition (PHaM) Risk Score

The primary independent variables of interest, such as age ≤ 24 months, the presence of gastrointestinal symptoms in the past week (e.g., inability to eat, nausea/vomiting, diarrhea, abdominal pain), fever > 39 °C, hospitalization due to lower respiratory tract infection, history of weight loss and reduced food intake, and disease severity (which was categorized as mild [grade 1], moderate [grade 2], or severe [grade 3] [8,17,18]), were included in the multivariate logistic regression model.

The final PHaM risk score (Table 2) was derived from five variables with seven potential predictive parameters, including the presence of gastrointestinal symptoms in the past week (no symptom, one symptom, or at least two symptoms); disease severity (mild; moderate to severe); presence of fever (no fever or ≤39 °C; >39 °C temperature); lower respiratory tract infection; and decrease in food intake. The scoring system assigns points to each risk factor proportional to its contribution to malnutrition risk. The score is calculated by adding individual item values ranging theoretically from a minimum value of 0 points to a maximum of 9 points. The minimum observed value was 0 points and the maximum recorded occurred at 9 points for both cohorts.

**Figure 1:**
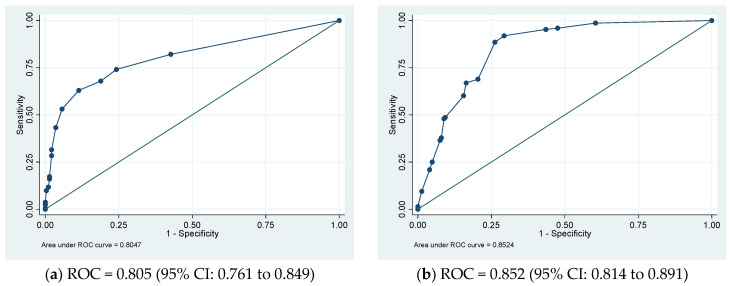
(**a**) Derivation and (**b**) validation sets’ receiver operating characteristic (ROC) curves for the pediatric hospital-acquired malnutrition (PHaM) risk score relative to hospital-acquired malnutrition.

**Figure 2:**
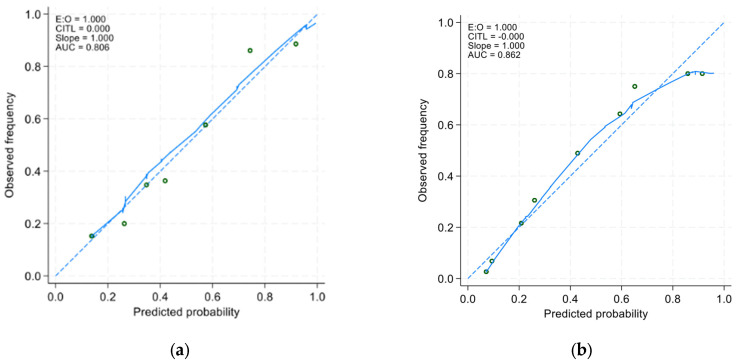
Calibration curves of (**a**) the derivative model and the (**b**) validation model. The *x*-axis represents the model-predicted frequency of hospital-acquired malnutrition, and the *y*-axis represents the actual frequency of hospital-acquired malnutrition.

In the derivative cohort, the model’s discriminatory ability, as indicated by AUROC, was 0.805 (95% CI: 0.761 to 0.849) (Figure 1a). The Youden index was employed to establish the optimal cutoff scores for PHaM risk score, yielding a value of ≥2.5 points for patients at high risk. Table 3 presented the performance indicators at various operating thresholds. The performance with a cutoff of ≥2.5 points exhibited a sensitivity of 63%, specificity of 88.6%, positive predictive value of 76.1%, negative predictive value of 80.6%, positive likelihood ratio of 5.53, and negative likelihood ratio of 0.42. The E:O ratio was 1.00 (95% CI: 0.90 to 1.10) (Figure 2a), and the Hosmer–Lemeshow test yielded a *p*-value of 0.551.

### 3.3. Model Validation

#### 3.3.1. The Internal Validation of the PHaM Risk Score

Internal validation of the risk score involved using 1000 bootstrap samples. The ROC was 0.805 (95% CI: 0.756 to 0.843), and the E:O ratio was 1.00 (95% CI: 0.89 to 1.10).

#### 3.3.2. The External Validation of the PHaM Risk Score

In the external validation group, the AUROC was 0.852 (95% CI 0.814 to 0.891) (Figure 1b), using a cutoff of ≥2.5 points resulted in improved sensitivity at 91.9% and negative predictive value at 93.0%, with a negative likelihood ratio of 0.11, compared to derivative cohort. However, specificity decreased to 70.7% with a positive predictive value of 67.3% PPV and a positive likelihood ratio of 3.13. The E:O ratio was 1.00 (95% CI: 0.90 to 1.10) (Figure 2b) and Hosmer-Lemeshow yielded *p*-value of 0.170.

### 3.4. Sensitivity Analysis

For our second sensitivity analysis we excluded 76 patients who received nutritional intervention upon or shortly (within 72 h) after admission resulting in only having data from 297 patients, which showed promising results, with the AUROC increasing to 0.927 (95% CI 0.898 to 0.955).

## 4. Discussion

Children’s nutritional status often declines during hospitalization, impacting clinical outcomes, growth, development, morbidity, and mortality. This decline can lead to delayed wound healing and recovery processes as well as increased healthcare costs [2,6]. While existing nutrition screening tools primarily focus on identifying malnutrition upon admission [19,20,21], it is essential to have a tool that can accurately assess nutritional risk throughout the entire hospitalization period in order to prompt appropriate interventions. Additionally, malnutrition upon admission largely depends on various factors that occur before admission such as anthropometric parameters, inadequate intake, or increased energy expenditure related to underlying medical condition, whereas hospital-acquired malnutrition occurs regardless of baseline nutritional status and is primarily linked to illness severity. This approach will help prevent further decline in nutritional status, enhancing clinical outcomes, and elevating the overall quality of care for these vulnerable patients.

In the development of a novel PHaM risk score, our study uncovered a substantial prevalence of hospital-acquired malnutrition at around 40% within the studied population. This significant finding underscores the crucial importance of efficient screening in pediatric healthcare, particularly in tertiary care settings, to promptly recognize the condition and provide management. Our risk assessment includes five clinical indicators and is straightforward, allowing non-specialists in nutrition to utilize it. The newly developed tool shows encouraging potential with an AUC of 0.805, signifying dependable predictive ability for identifying hospital-acquired malnutrition risk in pediatric patients.

Employing a cut-off score of 2.5 points to classify patients into HaM risk categories, the tool achieved a sensitivity of 63% and a specificity of 88.6%, suggesting it is capable of correctly identifying the majority of true negative cases. The PPV and NPV were both strong, at 76.1% and 80.6%, respectively, indicating the tool’s ability to give an accurate identifying at risk patients. Furthermore, the positive likelihood ratio of 5.53 suggests a significant increase in the odds of HaM being present when the test yields a positive result, whereas the negative likelihood ratio of 0.42 supports a decreased probability of HaM when the test is negative, reinforcing the tool’s utility in clinical settings. Overall, the new pediatric nutrition screening tool demonstrates good performance and reliability in identifying nutritional deterioration in hospitalized pediatric patients.

In the validation group, which was separate from the derivative cohort, enhancements across several metrics were noted. The AUC demonstrated an increase, signifying improved overall accuracy of the tool in this cohort. Sensitivity and NPV also showed an upturn, indicating the superior performance of the risk screening tool in identifying patients who are at risk for HaM and those who are not, respectively. Furthermore, the improvement in negative likelihood ratio in the validation group suggests a more effective means of ruling out HaM when the screening test is negative. However, differences in patient characteristics such as more severe disease upon admission, decreased dietary intake, and a higher percentage of undernourished and stunted individuals at increased risk of nutritional deterioration during hospitalization may have contributed to these changes. These improvements highlight the tool’s potential adaptability and reliability in various clinical settings, further advocating its utility in pediatric nutritional risk stratification. Overall, the new screening tool’s validation phase supports its effectiveness and adds credence to its implementation in practice for the timely identification of malnutrition in pediatric patients.

Several screening tools are available for identifying nutritional decline in hospitalized patients, employing various indicators such as clinical assessment, dietary intake, and anthropometric measurements. In 2000, Sermet-Gauddelus et al. [8] devised the Pediatric Nutritional Risk Score (PNRS), which encompasses reduced food intake, pain, and severity of pathologic condition, to create a scoring system ranging from 0 to 5 points. Tasci et al. [22] validated the Turkish version of PNRS and reported a sensitivity of 82.1%, specificity of 77.8%, PPV of 58.3%, and NPV of 92% using a cutoff point of 3 points. Recently developed screening tools such as the Pediatric Digital Scaled Malnutrition Risk screening Tool (PeDiSMART) [9] and the Pediatric Nutritional Screening Score (PNSS) [10], along with two simple questions related to food intake and poor weight gain [23], have emerged in upper- to middle-income countries. The PeDiSMART and PNSS tools involve anthropometric measurements that require dietitians or nutrition specialists for assessment. The two simple questions demonstrated lower sensitivity regarding significant body mass index z-score reduction. Integrating a combination of assessment modalities and involving multidisciplinary teams can enhance the accuracy and effectiveness of tools for identifying at-risk children and guiding targeted interventions to optimize nutritional outcomes during hospitalization. In clinical practice, especially those with limited resources, assessing the nutritional status of all inpatients thoroughly is challenging as it could strain daily medical care. The PNRS was similar to our PHaM score, which incorporates clinical parameters and does not require a dietitian, making it easier to use in resource-constrained settings. Additionally, the scoring system for the PHaM score has been developed based on regression coefficient, which is more accurate than risk ratio methods [14,15]. Despite this study involved secondary data analysis, the prospectively collected data allowed for the development of a comprehensive and clinically relevant screening tool. Our approach is consistent with existing tools in the literature but adds specificity by incorporating acute clinical indicators such as fever and lower respiratory tract infection. Future research should continue to validate and refine the PHaM tool, ensuring its applicability across diverse pediatric populations and clinical settings.

A significant concern was the tool’s ability to differentiate between acute and chronic conditions, given their differing pathophysiological mechanisms. Acute conditions, such as gastroenteritis, often result in rapid weight loss due to dehydration, while chronic conditions, such as inflammatory bowel disease or carcinoma, lead to more gradual nutritional decline. Our tool addresses these differences by categorizing patients based on the severity of their illness, with most acute, non-severe conditions classified as mild and more complex conditions, such as febrile neutropenia in cancer patients, classified as moderate. This stratification allows the tool to account for the varying degrees of nutritional risk associated with both acute and chronic conditions. Future research should focus on further refining these assessments to improve the tool’s accuracy across different clinical scenarios.

The pediatric hospital-acquired malnutrition (PHaM) screening tool was developed to create a comprehensive screening applicable across the entire pediatric age range, from young infants to adolescents. Our derivative and validation cohorts included children aged 1 month to 17.8 years. Compared to other available pediatric nutritional screening tools, such as the PeDiSMART [9], STRONGkids [19], and PNRS [8] tools, which also cover wide pediatric age ranges, the PHaM tool distinguished itself by including young infants (<12 months old), accounting for approximately 16.3–18.2% of our cohorts. While the PMST [24], PYMS [20], and STAMP [21] exclude younger infants, focusing on children over 1 or 2 years. These tools may avoid some challenges associated with assessing very young children but at the cost of missing early nutritional risks in this vulnerable population. To enhance the clinical utility of the PHaM tool, future studies should focus on refining age-specific criteria or factors, particularly for the neonatal (e.g., birth weight, gestational age, feeding pattern, and growth velocity) and adolescent (e.g., puberty, growth spurts, psychosocial factors, and dietary habits) age groups. By developing separate evaluation models for these distinct age groups, clinicians can better address the unique nutritional risks associated with each developmental stage, ensuring that all pediatric patients receive targeted and effective nutritional care during hospitalization.

The 72 h observation period was selected as the initial timeframe for assessing the risk of PHaM, based on the well-established phases of metabolic response to illness. The “ebb phase,” which occurs during the first 48–72 h after the onset of acute illness or injury, is characterized by reduced energy expenditure and catabolism. This is followed by the “flow phase,” where the body’s metabolic demands increase as it mounts a response to the illness [25,26,27,28]. As such, the 72 h mark is considered an optimal time point for identifying early signs of nutritional risk in hospitalized pediatric patients.

Despite this theoretical basis, we acknowledge that the 72 h observation period may not be sufficient to capture all potential nutritional complications, particularly in patients with complex or prolonged medical conditions. To address this limitation, the study protocol includes ongoing monitoring for patients initially classified as low-risk, with weekly reassessments until discharge. This approach allows for early identification of malnutrition while providing a safety net for detecting late-onset nutritional issues. We emphasize that the 72 h assessment should be part of a broader, continuous monitoring strategy, especially for high-risk patients, to ensure comprehensive nutritional care and prevent the development or worsening of malnutrition. Future studies should explore the effectiveness of this approach and consider whether extended or more frequent assessments can further enhance the early detection and management of hospital-acquired malnutrition.

In certain healthcare settings, such as tertiary care centers or facilities in developing countries with specialized disease-focused centers, there may be a need to calculate nutritional risk for specific diagnostic groups, including conditions such as cancer, congenital heart disease, and chronic lung disorders. While our study aimed to create a more generalizable screening tool by grouping patients based on disease severity, we acknowledge that diagnosis-specific risk assessments could offer more accurate and reliable evaluations. The PHaM screening tool was designed as an initial step to identify patients at risk or possibly at risk of nutritional deterioration. Our tool’s simplicity allows it to be used by a range of healthcare professionals, including nurses, internist, and general pediatricians. Specifically, the tool can be effectively utilized in secondary or tertiary care settings, where a positive screening result prompts detailed nutritional assessment along with appropriate nutritional intervention. Future iterations of the tool could incorporate advanced features that allow for more tailored risk calculations based on specific diagnoses, thus enhancing its precision and clinical utility.

The existing nutrition screening tools have certain limitations, presenting an opportunity to improve both the quality of screening and care. These include the following: (1) lack of well-established information on the effectiveness of these methods, in terms of sensitivity, specificity, validity, reliability, and cost-effectiveness; (2) lack of practical guidance on implementation; (3) potential complexity, time intensiveness, and invasiveness; (4) dependence on dietitians for data collection which hinders wider hospital-wide adoption by nursing or administration staff; and (5) reliance on anthropometric parameters that are not readily available. While our PHaM score showed moderate accuracy and ease of application in a tertiary care setting, further information is needed regarding cost-effectiveness as well as guidelines for managing at-risk patients. Additionally, the PHaM score’s generalizability to primary or secondary care levels needs further validation due to its original validation only within a tertiary care setting with complex disease severity and clinical conditions.

Our study aimed to develop a straightforward and practical nutritional screening tool applicable across the pediatric population under 18 years of age. While the tool shows promise, several limitations and considerations were identified.

Firstly, the physiological differences between age groups, particularly between a one-month-old newborn and an adolescent, are significant. We addressed this concern by performing a subgroup analysis for patients under 2 years and those 2 years and older, and we found that the significant parameters and the predictive abilities remained largely consistent across both age groups and with the original model. However, future studies should focus on refining the tool for specific age groups to enhance its accuracy and effectiveness. Additionally, the etiological characterization of gastrointestinal symptoms or lower respiratory tract infection are very important for the assessment of disease-related nutritional status. Our PHaM score was designed to identify patients at high risk, after which they will undergo a comprehensive nutritional assessment. During the assessment, the specific etiology of the disease will be thoroughly determined, allowing a tailored and appropriate management plan.

Secondly, our study did not include laboratory parameters due to their lack of statistical significance in differentiating patients who developed hospital-acquired malnutrition from those who did not in our previous studies (*p*-value > 0.25). Moreover, in primary care settings, laboratory testing may be limited and is not typically included in basic assessments. Our screening tool relies on easily accessible clinical information, making it practical for use by a range of healthcare professionals, including nurses, internists, and general pediatricians. In secondary or tertiary care settings, a positive screening result would lead to a more detailed nutritional assessment and intervention by dietitians or pediatricians with expertise in nutritional support.

We also recognize the importance of psychological, psychiatric, and social factors influencing under-treatment. While our focus was on clinical parameters, future research should incorporate these additional factors to enhance the comprehensiveness and clinical applicability of the screening tool.

Moreover, the relatively short observation period of our study could lead to potential misinterpretation of results due to the variability in disease etiologies. To mitigate this limitation, we performed a multicenter study to capture a diverse patient population, enhancing the generalizability and robustness of our findings. We recommend future studies with extended observation periods to provide additional valuable data and further refine the screening tool.

Lastly, the PHaM score is a diagnostic prediction tool designed to identify early stages of nutritional deterioration in hospitalized pediatric patients. It effectively predicts current nutritional risk shortly after admission but is not intended to predict long-term outcomes or the exact timing of safe discharge from the hospital [29]. For patients who test negative on the initial screening, we will reevaluate their nutritional status every 7 days until discharge. This ongoing monitoring ensures that any emerging nutritional risks are promptly identified and addressed, thereby maintaining patient safety and promoting optimal recovery.

## 5. Conclusions

This study contributes to our knowledge of nutritional deterioration and provide practical solutions to improve patient outcomes. A new risk score, called the “PHaM” score, could be utilized in tertiary care hospitals, presenting opportunities to enhance care for inpatients by improving detection of pediatric hospital-acquired malnutrition care in terms of the nutritional aspect, which includes early detection of pediatric hospital-acquired malnutrition and awareness of this condition. However, further research and validation studies are needed to assess generalizability and clinical utility of this tool and approaches in diverse pediatric populations and clinical settings, ultimately advancing nutritional assessment and management practice, and establishing a specific coding system for this condition as well as for reimbursement.

## Figures and Tables

**Table 1:** Demographic and clinical characteristics of study participants in the model development cohort and validation cohort.

Variables	Patients in Derivative Cohort [N = 444], n (%)	Patients in Validation Cohort [N = 373], n (%)	*p*-Value
Sex			0.002
Male	265 (59.7%)	181 (48.5%)	
Female	180 (40.3%)	192 (51.5%)	
Age			0.864
Age ≤ 1 year	72 (16.2%)	68 (18.2%)	
Age 1–5 years	163 (36.7%)	129 (34.6%)	
Age 5–12 years	129 (29.1%)	109 (29.2%)	
Age 12 years	80 (18.0%)	67 (18.0%)	
Median (IQR) (years)	4.3 (8.7)	4.7 (9.4)	0.484
Underlying disease *	322 (72.5%)	220 (59.0%)	<0.001
Disease severity			<0.001
Mild	355 (80.0%)	184 (49.3%)	
Moderate	82 (18.5%)	169 (45.3%)	
Severe	7 (1.5%)	20 (5.4%)	
History of weight loss	105 (23.6%)	65 (17.4%)	0.029
Decreased food intake	152 (34.2%)	169 (45.3%)	0.001
Nutritional status on admission			
HAZ ^#^	−0.91 (2.17)	−1.14 (2.26)	0.003
WAZ ^#^	−0.76 (1.95)	−0.96 (2.21)	0.004
WHZ ^#^	−0.34 (2.08)	−0.54 (2.10)	0.054
BMIZ ^#^	−0.51 (2.24)	−0.53 (2.26)	0.499
Wasting (WHZ < −2)	69 (15.5%)	74 (19.8%)	0.107
Stunting (HAZ < −2)	111 (25.0%)	119 (31.9%)	0.029
Underweight (WAZ < −2)	93 (20.9%)	111 (29.8%)	0.003
Obesity (BMIZ > 2)	26 (5.6%)	26 (7.0%)	0.515
Nosocomial infection, number	85 (19.1.%)	89 (23.9%)	0.100
Pneumonia	22 (4.9%)	38 (10.2%)	
Surgical wound infection	11 (2.5%)	9 (2.4%)	
Bloodstream infection or CLABSI	21 (4.7%)	18 (4.8%)	
Diarrhea/AAC	6 (1.4%)	4 (1.1%)	
Clinical sepsis	19 (4.3%)	14 (3.8%)	
Urinary tract infection	6 (1.4%)	6 (1.6%)	
Length of hospital stay, days ^#^	5 (4)	7 (10)	<0.001
Nutritional intervention	47 (10.6%)	104 (27.9%)	<0.001

* One patient could have more than one underlying disease. ^#^ Data are expressed as median (interquartile range). AAC: antibiotic-associated colitis; BMIZ: body mass index-for-age z-score; CLABSI: central line-associated blood stream infection; HAZ: length/height-for-age z-score; WAZ: weight-for-age z-score; WHZ: weight-for-length/weight z-score.

**Table 2:** Final multivariable analysis for hospital-acquired malnutrition risk in derivative cohort.

Patient Characteristics	Coefficient	Odds Ratio (95% CI)	*p*-Value	Score Value
Gastrointestinal symptom			<0.001	
No symptom		1		0
1 symptom	1.201	3.325 (1.815 to 6.088)		1.5
≥2 symptoms	1.235	3.440 (1.304 to 9.076)		1.5
Disease severity			<0.001	
Mild		1		0
Moderate to severe	1.444	4.237 (2.374 to 7.563)		2
Fever			<0.001	
No fever or fever ≤ 39 °C		1		0
Fever > 39 °C	2.700	14.828 (5.811 to 37.836)		3.5
Lower respiratory tract infection			0.024	
No		1		0
Yes	0.767	2.153 (1.108 to 4.186)		1
Decreased food intake			0.001	
No		1		0
Yes	0.817	2.264 (1.379 to 3.716)		1
Constant	−1.832			

**Table 3:** Performance metrics at optimal operating point.

Cut-Off Score	Sensitivity (95% CI)	Specificity (95% CI)	Youden’s Index	PPV (95% CI)	NPV (95% CI)	LR+ (95% CI)	LR− (95% CI)
1	82.1% (75.3% to 87.7%)	57.3% (51.3% to 63.2%)	0.394	52.6% (46.2% to 58.9%)	84.7% (78.8% to 89.5%)	1.92 (1.65 to 2.24)	0.31 (0.22 to 0.44)
1.5	74.1% (66.6% to 80.6%)	75.8% (70.4% to 80.7%)	0.499	63.8% (56.5% to 70.7%)	83.5% (78.4% to 87.9%)	3.06 (2.44 to 3.84)	0.34 (0.26 to 0.45)
2	67.9% (60.1% to 75.0%)	81.1% (76.1% to 85.5%)	0.490	67.5% (59.7% to 74.6%)	81.4% (76.4% to 85.8%)	3.60 (2.76 to 4.69)	0.40 (0.31 to 0.50)
2.5	63.0% (55.0% to 70.4%)	88.6% (84.3% to 92.1%)	0.516	76.1% (68.0% to 83.1%)	80.6% (75.7% to 84.8%)	5.53 (3.91 to 7.82)	0.42 (0.34 to 0.51)
3	53.1% (45.1% to 61.0%)	94.3% (90.9% to 96.7%)	0.474	84.3% (75.8% to 90.8%)	77.7% (72.9% to 82.0%)	9.32 (5.67 to 15.3)	0.50 (0.42 to 0.59)
3.5	43.2% (35.5% to 51.2%)	96.4% (93.6% to 98.3%)	0.396	87.5% (78.2% to 93.8%)	74.7% (69.9% to 79.1%)	12.14 (6.44 to 22.88)	0.59 (0.51 to 0.67)
4	31.5% (24.4% to 39.2%)	97.9% (95.4% to 99.2%)	0.294	89.5% (78.5% to 96.0%)	71.2% (66.4% to 75.7%)	14.74 (6.47 to 33.59)	0.70 (0.63 to 0.78)
4.5	28.4% (21.6% to 36.0%)	97.9% (95.4% to 99.2%)	0.263	88.5% (76.6% to 95.6%)	70.3% (65.5% to 74.8%)	13.30 (5.81 to 30.45)	0.73 (0.66 to 0.81)
5	17.3% (11.8% to 24.0%)	98.6% (96.4% to 99.6%)	0.159	87.5% (71.0% to 96.5%)	67.4% (62.6% to 71.9%)	12.14 (4.34 to 34.00)	0.84 (0.78 to 0.90)
5.5	16.1% (10.8% to 22.6%)	98.6% (96.4% to 99.6%)	0.147	86.7% (69.3% to 96.2%)	67.1% (62.3% to 71.6%)	11.27 (4.01 to 31.73)	0.85 (0.80 to 0.91)
6	11.7% (7.2% to 17.7%)	98.9% (96.9% to 99.8%)	0.106	86.4% (65.1% to 97.1%)	66.0% (61.3% to 70.5%)	10.99 (3.30 to 36.55)	0.89 (0.84 to 0.94)
6.5	9.9% (5.8% to 15.5%)	99.6% (98.0% to 100.0%)	0.095	94.1% (71.3% to 99.9%)	65.7% (61.0% to 70.2%)	27.75 (3.71 to 207.34)	0.90 (0.86 to 0.95)

CI: confidence interval; LR+: positive likelihood ratio; LR−: negative likelihood ratio; NPV: negative predictive value; PPV: positive predictive value.

## Data Availability

The data presented in this study are available on request from the first author (S.S.). The data are not publicly available due to ethical considerations and patient confidentiality.

## Supplementary Materials

The following supporting information can be downloaded at: https://www.mdpi.com/article/10.3390/nu16172898/s1, Table S1: Underlying Medical Conditions of Participants; Supplementary Materials S2: Definition of Nutritional Intervention in This Study.

## References

[1] Viana Alves M.d.F., Cruvel J.M.d.S., Coutinho M.A., Sousa M.M.B., Barbosa E.C.B., Pires B.R.F. (2023). Hospital-Acquired Undernutrition and Associated Factors in Children and Adolescents Admitted to a Tertiary Care Hospital. J. Hum. Nutr. Diet..

[2] Campanozzi A., Russo M., Catucci A., Rutigliano I., Canestrino G., Giardino I., Romondia A., Pettoello-Mantovani M. (2009). Hospital-Acquired Malnutrition in Children with Mild Clinical Conditions. Nutrition.

[3] Hecht C., Weber M., Grote V., Daskalou E., Dell’Era L., Flynn D., Gerasimidis K., Gottrand F., Hartman C., Hulst J. (2015). Disease Associated Malnutrition Correlates with Length of Hospital Stay in Children. Clin. Nutr..

[4] Hwang E.H., Park J.H., Chun P., Lee Y.J. (2016). Prevalence and Risk Factors for the Weight Loss during Hospitalization in Children: A Single Korean Children’s Hospital Experience. Pediatr. Gastroenterol. Hepatol. Nutr..

[5] Sean Quadros D.-R., Kamenwa R., Akech S., M Macharia W. (2018). Hospital-Acquired Malnutrition in Children at a Tertiary Care Hospital. S. Afr. J. Clin. Nutr..

[6] Saengnipanthkul S., Chongviriyaphan N., Densupsoontorn N., Apiraksakorn A., Chaiyarit J., Kunnangja S., Wongpratoom S., Papakhee S., Det-amnatkul W., Monwiratkul J. (2021). Hospital-Acquired Malnutrition in Paediatric Patients: A Multicentre Trial Focusing on Prevalence, Risk Factors, and Impact on Clinical Outcomes. Eur. J. Pediatr..

[7] Bélanger V., McCarthy A., Marcil V., Marchand V., Boctor D.L., Rashid M., Noble A., Avinashi V., Davidson B., Groleau V. (2019). Assessment of Malnutrition Risk in Canadian Pediatric Hospitals: A Multicenter Prospective Cohort Study. J. Pediatr..

[8] Sermet-Gaudelus I., Poisson-Salomon A.S., Colomb V., Brusset M.C., Mosser F., Berrier F., Ricour C. (2000). Simple Pediatric Nutritional Risk Score to Identify Children at Risk of Malnutrition. Am. J. Clin. Nutr..

[9] Karagiozoglou-Lampoudi T., Daskalou E., Lampoudis D., Apostolou A., Agakidis C. (2015). Computer-Based Malnutrition Risk Calculation May Enhance the Ability to Identify Pediatric Patients at Malnutrition-Related Risk for Unfavorable Outcome. JPEN J. Parenter. Enter. Nutr..

[10] Lu L., Mao X., Sheng J., Huang J., Wang Y., Tang Q., Cai W. (2018). Development and Validation of a Pediatric Nutritional Screening Score (PNSS) for Hospitalized Children. Asia Pac. J. Clin. Nutr..

[11] Ferguson M., Capra S., Bauer J., Banks M. (1999). Development of a Valid and Reliable Malnutrition Screening Tool for Adult Acute Hospital Patients. Nutrition.

[12] WHO Anthro Survey Analyser and Other Tools. https://www.who.int/tools/child-growth-standards/software.

[13] World Health Organization (2002). Prevention of Hospital-Acquired Infections: A Practical Guide.

[14] Mehta H.B., Mehta V., Girman C.J., Adhikari D., Johnson M.L. (2016). Regression Coefficient-Based Scoring System Should Be Used to Assign Weights to the Risk Index. J. Clin. Epidemiol..

[15] Sullivan L.M., Massaro J.M., D’Agostino Sr R.B. (2004). Presentation of Multivariate Data for Clinical Use: The Framingham Study Risk Score Functions. Stat. Med..

[16] Youden W.J. (1950). Index for Rating Diagnostic Tests. Cancer.

[17] Riley R.D., Ensor J., Snell K.I.E., Harrell F.E., Martin G.P., Reitsma J.B., Moons K.G.M., Collins G., Smeden M. (2020). van Calculating the Sample Size Required for Developing a Clinical Prediction Model. BMJ.

[18] Ensor J. (2023). Pmsampsize: Sample Size for Development of a Prediction Model. https://cran.r-project.org/web/packages/pmsampsize/pmsampsize.pdf.

[19] Huysentruyt K., Alliet P., Muyshont L., Rossignol R., Devreker T., Bontems P., Dejonckheere J., Vandenplas Y., De Schepper J. (2013). The STRONGkids Nutritional Screening Tool in Hospitalized Children: A Validation Study. Nutrition.

[20] Gerasimidis K., Macleod I., Maclean A., Buchanan E., McGrogan P., Swinbank I., McAuley M., Wright C.M., Flynn D.M. (2011). Performance of the Novel Paediatric Yorkhill Malnutrition Score (PYMS) in Hospital Practice. Clin. Nutr..

[21] McCarthy H., Dixon M., Crabtree I., Eaton-Evans M.J., McNulty H. (2012). The Development and Evaluation of the Screening Tool for the Assessment of Malnutrition in Paediatrics (STAMP©) for Use by Healthcare Staff. J. Hum. Nutr. Diet..

[22] Taşcı O., Bekem Soylu Ö., Kıran Taşcı E., Eser E., Oruçoğlu B., Günay İ. (2020). Validity and Reliability Analysis of the Turkish Version of Pediatric Nutritional Risk Score Scale. Turk. J. Gastroenterol..

[23] White M.S., Ziemann M., Doolan A., Song S.Q., Bernard A. (2019). A Simple Nutrition Screening Tool to Identify Nutritional Deterioration in Long Stay Paediatric Inpatients: The Paediatric Nutrition Rescreening Tool (PNRT). Clin. Nutr. ESPEN.

[24] Thomas P.C., Marino L.V., Williams S.A., Beattie R.M. (2016). Outcome of Nutritional Screening in the Acute Paediatric Setting. Arch. Dis. Child..

[25] Preiser J.-C., Ichai C., Orban J.-C., Groeneveld A.B.J. (2014). Metabolic Response to the Stress of Critical Illness. Br. J. Anaesth..

[26] Biffl W.L., Moore E.E., Haenel J.B. (2002). Nutrition Support of the Trauma Patient. Nutrition.

[27] Cuthbertson D.P., Angeles Valero Zanuy M.A., León Sanz M.L. (2001). Post-Shock Metabolic Response. 1942. Nutr. Hosp..

[28] Hasenboehler E., Williams A., Leinhase I., Morgan S.J., Smith W.R., Moore E.E., Stahel P.F. (2006). Metabolic Changes after Polytrauma: An Imperative for Early Nutritional Support. World J. Emerg. Surg..

[29] Iwagami M., Matsui H. (2022). Introduction to Clinical Prediction Models. Ann. Clin. Epidemiol..

