# Supplementary material for: Development and Validation of a Pediatric Hospital-Acquired Malnutrition (PHaM) Risk Score to Predict Nutritional Deterioration in Hospitalized Pediatric Patients: A Secondary Analysis Based on a Multicenter Prospective Cohort Study
Source: Nutrients. 2024 Aug 29;16(17):2898. doi: 10.3390/nu16172898 (PMC11396899; doi:10.3390/nu16172898)
Supplement: Supplementary file 1 [file nutrients-16-02898-s001.zip › nutrients-3082787-supplementary.pdf]

Supplementary Table S1 Underlying Medical Conditions of Participants

Variables	Derivative cohort (n=444)	Validation cohort (n=373)
Having underlying medical conditions	322 (72.5%)	220 (59.0%)
Cancer	89 (27.6%)	47 (21.4%)
Chronic cardiac disease	42 (13.0%)	20 (9.1%)
Epilepsy	35 (10.9%)	21 (9.4%)
Prematurity (corrected age 2 years)	20 (6.2%)	18 (8.2%)
Biliary atresia	18 (5.6%)	12 (5.5%)
Autoimmune rheumatic diseases	16 (5.0%)	9 (4.1%)
Asthma	16 (5.0%)	12 (5.5%)
Global developmental delay	15 (4.7%)	10 (4.5%)
Metabolic syndrome	13 (4.0%)	9 (4.1%)
Chronic kidney disease	12 (3.7%)	10 (4.5%)
Chronic liver disease	11 (3.4%)	6 (2.7%)
Chronic lung disease	9 (2.8%)	10 (4.5%)
Oromotor dysfunction	9 (2.8%)	12 (5.5%)
Intestinal malabsorption	8 (2.5%)	12 (5.5%)
Food allergy/cow's milk protein allergy	5 (1.6%)	5 (2.3%)
Bronchopulmonary dysplasia	2 (0.6%)	4 (1.8%)
Depression/mood disorder	2 (0.6%)	3 (1.4%)

Supplementary Materials S2. Definition of Nutritional Intervention in This Study

In our study, nutritional support includes a range of interventions designed to meet the individual needs of the patients. These interventions include:

1. **Dietary Modifications:** We offer diets customized to the patient's specific needs, such as specific calorie requirements, protein quantity and quality, diabetic diets, and high-protein diets. These diets are planned and monitored by dietitians to ensure they meet the nutritional needs of the patients.
2. **Oral Nutritional Supplements:** For patients who can consume food orally but require additional nutritional support, we provide oral nutritional supplements with medical formulas. These supplements are designed to ensure that patients receive the necessary nutrients to support their recovery and overall health.
3. **Enteral Feeding:** For patients who cannot meet their nutritional needs through oral intake alone, enteral feeding is provided. This includes tube feeding, where a feeding tube is placed into the stomach or small intestine to deliver nutrient-rich formulas directly.
4. **Parenteral Nutrition:** In cases where enteral feeding is not possible or sufficient, parenteral nutrition is administered. This involves delivering nutrients directly into the bloodstream through an intravenous (IV) line. Parenteral nutrition is carefully monitored to ensure it meets the patient's nutritional requirements without causing complications.
